# Modulation of Glucose Consumption and Uptake in HepG2 Cells by Aqueous Extracts from the Coelomic Fluid of the Edible *Holothuria tubulosa* Sea Cucumber

**DOI:** 10.3390/biology13060378

**Published:** 2024-05-25

**Authors:** Giulia Abruscato, Roberta Tarantino, Manuela Mauro, Roberto Chiarelli, Aiti Vizzini, Vincenzo Arizza, Mirella Vazzana, Claudio Luparello

**Affiliations:** 1 Dipartimento di Scienze e Tecnologie Biologiche Chimiche e Farmaceutiche (STEBICEF), Università di Palermo, 90128 Palermo, Italy; giulia.abruscato@unipa.it (G.A.); roberta.tarantino@community.unipa.it (R.T.); manuela.mauro01@unipa.it (M.M.); roberto.chiarelli@unipa.it (R.C.); aiti.vizzini@unipa.it (A.V.); vincenzo.arizza@unipa.it (V.A.); mirella.vazzana@unipa.it (M.V.); 2 National Biodiversity Future Center (NBFC), 90133 Palermo, Italy

**Keywords:** echinoderm, coelomic fluid extract, anti-diabetic, GLUT2, GLUT4, IRS1, AKT, HNF1α, PAS staining, 2-NBDG

## Abstract

**Simple Summary:**

*Holothuria tubulosa* is a widespread edible Mediterranean species of sea cucumber. In an attempt to identify new compounds that might exert an anti-diabetic effect, the aqueous extract from the coelomic fluid (CFE) of *H. tubulosa* was prepared and tested for its extracellular glucose-lowering activity on hepatic cells in culture. Here, we have identified some cellular and molecular aspects of the beneficial effect of the CFE that stimulates glucose consumption/uptake and glycogen storage inducing the increased synthesis and translocation to the cell surface of the glucose transporters GLUT-2 and -4. These marine-derived natural material is worth further exploration aimed at developing novel alternative agents against diabetes mellitus and beneficial supplements for the formulation of functional food.

**Abstract:**

The cell-free aqueous extract from the coelomic fluid of *Holothuria tubulosa* was prepared and examined for its glucose-lowering effect on HepG2 cells in vitro. In particular, employing a combination of cytochemical, flow cytometric, PCR, and protein blot techniques, we evaluated its role on glucose internalization and storage and on the upregulation and surface translocation of the two glucose transporters GLUT-2 and -4. The changes in expression, synthesis, and/or activation of the *GLUT2*-related transcription factor hepatocyte nuclear factor-1 alpha (HNF1α) and the GLUT-4-translocation regulatory factors insulin receptor substrate-1 (IRS-1) and AKT were also studied. Our results showed the improved glucose response by HepG2 cells, leading to an evident increase in glucose consumption/uptake and glycogen storage upon exposure. Moreover, the extract induced molecular reprogramming involving the upregulation of (i) *IRS1* gene expression, (ii) the transcription and translation levels of HNF1α, AKT, and GLUT-4, (iii) the phosphorylation level of AKT, (iv) the synthesis of GLUT-2 protein, and (v) the translocation of GLUT-2 and -4 transporters onto the plasma membrane. Cumulatively, our results suggest that the coelomic fluid extract from *H. tubulosa* can be taken into consideration for the development of novel treatment agents against diabetes mellitus.

## 1. Introduction

It is widely acknowledged that seas and oceans, which cover three-quarters of the Earth’s surface, represent nature’s untapped treasure chest. The hosted animals, plants, and microorganisms, in fact, contribute to creating a reservoir of natural medicines, food additives, and cosmetics via their primary and secondary metabolites, many of which still await discovery. Such unique molecules, which regulate very different biological activities, play an important ecological role in the processes of chemical defense, communication, and reproduction in organisms. In fact, they implement the adaptation mechanisms necessary for the survival of species in the heterogeneous, and often extreme, conditions of marine habitats. The development and application of marine biodiscovery [1] has brought to light several natural compounds able to interfere with the onset and progress of numerous pathogenic diseases and to exert a wide range of therapeutic effects against, e.g., cancer, inflammation, and infections [2,3,4,5,6].

Among marine invertebrates, edible sea cucumbers that belong to the class Holothuroidea of the phylum Echinodermata have been proven to exert several beneficial effects on human health via the wide range of bioactive molecules present in their different body parts that are endowed with diversified properties (e.g., antioxidant, neuroprotective, and antitumoral) [7,8]. *Holothuria tubulosa* (Gmelin, 1788) is a widespread edible Mediterranean species that lives on muddy rocks or sandy bottoms within meadows of *Posidonia oceanica*. It displays a pale to reddish brown surface covered by several dark long conical papillae and three lines of mobile tube-feet along the ventral face [9]. Some literature reports have been focused on the nutritional and pharmacological values of the extracts and isolated compounds obtained from this sea cucumber. A study on experimentally edematous mice demonstrated the anti-inflammatory role of a methanol extract from *H. tubulosa* through the partial inhibition of cyclooxygenase-2 activity [10]. The low M.W. fucoidan isolated from the body wall of *H. tubulosa* was shown to attenuate the metabolic disease-related inflammatory reaction both in vitro and in vivo. The conspicuous fatty acid content of the tegument of *H. tubulosa*, apart from representing an important nutritional quality, was proven to exert in vitro antioxidant and free radical-scavenging effects [11]. In addition, total extracts of the body of this sea cucumber promoted apoptosis-based cytotoxicity against cancer cells, and the enzymatic hydrolysates from the body wall were also revealed as a source of angiotensin-converting enzyme (ACE) inhibitory peptides [12,13]. Moreover, its coelomocytes, the immune mediator cells, were shown to be the source of the antimicrobial peptides holothuroidin-1 and -2 [14].

In echinoderms, coelomic fluid is a significant portion of their body mass. It provides a transport- and immune response-designed pseudo-vascular system and hosts a complex mixture of soluble molecules, mostly proteins, secreted constitutively by different parts of the invertebrate’s body. Using the cell-free water extract from the coelomic fluid (CFE) of *H. tubulosa*, we have previously demonstrated its in vitro anti-breast and liver cancer effects. In fact, when supplied to the cultures of cancer cells, the preparation inhibited cell viability and proliferation by impairing cell cycle progress, locomotion, mitochondrial activity, reactive oxygen species (ROS) production, and autophagic flux, in some cases stimulating apoptotic death. A comprehensive proteomic analysis of the CFE complemented the biological data identifying a subgroup of proteins potentially responsible for anti-liver cancer activity [15,16].

Diabetes mellitus is a metabolic disorder characterized by chronic hyperglycemia due to the inability to produce sufficient insulin or to use it efficiently. It is a serious threat to human health considering that, worldwide, more than a half billion people are living with diabetes, 96% with the mellitus type, and that the number is expected to more than double in the next 30 years [17]. Numerous pharmacological resources are available to help to control blood glucose levels, but many side effects are associated with the use of these drugs. On the other hand, as reviewed in [18,19], the development of anti-diabetic compounds from natural origins has attracted much attention as a safe, effective, available, and low-cost alternative, holding promise for its potential beneficial effects, although taking into account that rigorous scientific validation is essential for their medicinal use.

The health-improving benefits of the regular intake of food containing bioactive substances have long been recognized, prompting studies for their application in the biomedical and biotechnological fields. Since to our knowledge there is no information on the potential anti-diabetic activity of the CFE of *H. tubulosa*, in the present study we investigated whether the preparation could exert in vitro glucose-lowering effects. Liver is the main organ where glucose metabolism and storage take place and the main target for the molecular alterations of glucose uptake and utilization. For this reason, HepG2 liver cancer cells were chosen as the study model system, since they express many differentiated hepatic functions, including glycogen storage and insulin signalization [20]. Here, employing a combination of cytochemical, flow cytometric, PCR, and protein blot techniques, we examined the role of the CFE in glucose consumption, internalization, and accumulation and in the upregulation and surface translocation of the two glucose transporters GLUT-2 and -4 [21]. The changes in expression, synthesis, and/or activation of the *GLUT2*-related transcription factor hepatocyte nuclear factor-1 alpha (HNF1α) [22] and the GLUT-4-translocation regulatory factors insulin receptor substrate-1 (IRS-1) and AKT [23,24] were also studied.

## 2. Materials and Methods

### 2.1. Preparation and Analysis of the CFE

The CFE from *H. tubulosa* was obtained as already reported by Luparello et al. [15,16]. Briefly, healthy adult specimens fished in the Gulf of Palermo (Sicily, Italy) were kept in aquarium for acclimation until their use. Once collected by bleeding, their coelomic fluid was centrifuged to separate the cell-free preparation from the coelomocytes and lyophilized. After resuspension in the minimum volume of distilled water, the protein concentration was determined through fluorometric assay using the Qubit Protein Assay Kit (ThermoFisher, Waltham, MA, USA). Aliquots of the preparation were subjected to proteomic analysis, and the partial results have been reported by Luparello et al. [16].

### 2.2. Cell Culture and Treatment

HepG2 liver cancer cells were routinely cultured in high glucose–DMEM medium (D6429; Sigma, St. Louis, MO, USA) with the addition of an antibiotic/antimycotic solution (A5955; Sigma) and 10% fetal calf serum (F4135; Sigma) in a humidified incubator at 37 °C and a 5% CO_2_ atmosphere. In light of the previous results of viability assay [16], the maximum sublethal CFE concentration of 2 μg/mL was selected for the subsequent experiments.

### 2.3. PAS Staining

HepG2 cells were seeded at a concentration of 88,000 cells/well in 6-well plates and, once subconfluent, the treatment with either the CFE, with or without the supplement of 10^−7^ M insulin (Santa Cruz Biotechnology, Heidelberg, Germany), or the sole 10^−7^ M insulin was applied for 24 h. Untreated cells were used as controls. The assessment of glycogen accumulation through PAS staining was carried out according to Donato et al. [20]. Essentially, control and treated cells were fixed with 4% paraformaldehyde in PBS (Santa Cruz Biotechnology), covered with 0.5% periodic acid solution (Santa Cruz Biotechnology), and finally treated with the Schiff’s reagent (Santa Cruz Biotechnology). The appearance of the stained glycogen granules was observed under the light microscope and photographed.

### 2.4. Extracellular Glucose Determination

HepG2 cells were seeded at a concentration of 40,000 cells/well in 24-well plates and, once subconfluent, the treatment with either the CFE, with or without the supplement of 10^−7^ M insulin (Santa Cruz Biotechnology), or the sole 10^−7^ M insulin was applied for 24 h. The extracellular glucose was measured as reported by Zhang et al. [25] and Cruz-Bermúdez et al. [26]. Essentially, at the end of the treatment the media were removed and diluted 1:1 with sterile distilled water before the measurement of their glucose concentration, which was performed in triplicate using a blood glucose meter (Glucomen Areo 2k, Menarini Diagnostics, Firenze, Italy) and single-use test strips (Glucomen Areo Sensor, Menarini Diagnostics). The media from untreated cell cultures were used as controls.

### 2.5. Flow Cytometric Assays for Glucose Uptake Determination and GLUT-2 and -4 Exposure on the Plasma Membrane

Flow cytometric analyses were performed using the FACScanto instrument (BD Biosciences, Franklin Lakes, NJ, USA) with the evaluation of 10,000 single-cell events. The obtained fcs files were analyzed with the Floreada tool available online at https://floreada.io (accessed on 20 September and 7 December 2023). The cell debris, which displayed low FSC values, were excluded from every analysis by gating in the FSC vs. SSC plot. HepG2 cells were seeded at the concentration of 88,000 cells/well in 6-well plates and, once subconfluent, the treatment with either the CFE or 10^−7^ M insulin (Santa Cruz Biotechnology) was applied for 24 h. Untreated cells were used as controls.

The glucose uptake was evaluated as reported by Csepregi et al. [27]. Essentially, at the end of the incubation, the media were discarded and replaced with Ca^++^/Mg^++^-containing PBS, and the cells were exposed to the fluorescent glucose analog 2-(N-(7-nitrobenz-2-oxa-1,3-diazol-4-yl)amino)-2-deoxyglucose (2-NBDG; Peptide Institute, Osaka, Japan) for 1 h. Subsequently, the cells were trypsinized, and the amount of 2-NBDG taken up by the cells was measured by flow cytometry in the fluorescein isothiocyanate (FITC) channel after addition of propidium iodide (PI) to identify the dead cell population. A control preparation without the addition of 2-NBDG was included in the analysis.

The quantitative evaluation of the exposure of GLUT-2 and -4 carriers on the plasma membrane was performed through immunostaining following the protocols of Koshy et al. [28] and Bruzzone et al. [29]. In brief, at the end of the incubation, the cells were trypsinized, washed with ice-cold PBS, and incubated for 20 min. in the cold with either GLUT-2 or GLUT-4 polyclonal antibodies (bs-10379R-TR and bs-0384R-TR, Bioss, Boston, MA, USA; working dilution 1:100) dissolved in 3% BSA-containing PBS. The incubation with the FITC-conjugated secondary antibody (AP132F, Sigma, working dilution 1:80) was performed for further 20 min. and, after an additional washing, the cell preparations were immediately submitted to flow cytometry for the data acquisition. An isotype control was included in the analysis.

### 2.6. Conventional and Quantitative Real-Time Polymerase Chain Reaction (qRT-PCR)

The analysis of gene expression was performed through conventional and qRT-PCR [30]. Essentially, the total RNAs from control and treated cells were isolated using the PureLink RNA Mini kit (ThermoFisher) performing the on-column DNase treatment using the PureLink DNase set (ThermoFisher), as recommended by the manufacturer. In total, 500 ng of RNA was reverse transcribed using the RevertUP^TM^ II Reverse transcriptase kit (Biotechrabbit, Berlin, Germany) and random hexamer primers according to the manufacturer’s instructions.

The conventional PCR analysis was carried out using 2.5 μM of the appropriate sense and antisense primers (see Table 1), 1 U BIOTAQ^TM^ DNA polymerase (Meridian Bioscience, Cincinnati, OH/USA)/μL, 200 μM each of dNTPs, and 1 μL of the cDNA template obtained from the total RNA. The thermal cycle used was a denaturation step of 94 °C for 2 min, followed by 33 cycles of 94 °C for 1 min, the appropriate annealing temperature for 1 min, and 72 °C for 1 min. The final extension of the product was performed for 5 min at 72 °C. The PCR products were analyzed by 1% agarose gel electrophoresis and visualized through Gel Red staining (Biotium, Fremont, CA, USA) under UV light. The quantitative analysis of the bands, when performed, was carried out with the ImageJ software with the normalization set to *ACTB* band intensity, which was used as the internal control. The differential expression of *GLUT2*, *GLUT4*, and *HNF1A* genes was further checked through the SYBR-Green-based qRT-PCR using the SYBR-Green qPCR MasterMix (MedChem Express, Monmouth Junction, NJ, USA) in an Applied Biosystems 7500 Real-Time PCR system, as previously reported by Abruscato et al. [30]. The specificity of the amplification was checked by real-time PCR melting analysis, and the 2^−ΔΔCt^ method was used to quantitate the samples, as described in the Applied Biosystems Use Bulletin N.2 (P/N 4303859). The normalized quantification of the expression levels of each target gene was performed by using *ACTB* as the internal control and expressed as target/reference ratio.

### 2.7. Western Blotting

The Western blot analysis was performed as described elsewhere [30]. Essentially, control and treated HepG2 cells were collected and lysed in a buffer containing 7 M Urea, 2% CHAPS, and 10 mM DTT, supplemented with a protease inhibitor cocktail (Sigma). Equal amounts of the proteins were separated by 13% sodium dodecyl sulphate-polyacrylamide gel electrophoresis (SDS-PAGE), and then the protein bands were transferred to the nitrocellulose membranes. The rabbit primary antibodies used to probe the blots were anti-GLUT2 (bs-10379R-TR, Bioss, Boston, MA, USA; working dilution 1:500), anti-GLUT4 (bs-0384R-TR, Bioss; working dilution 1:500), anti-HNF1α (PAG775Hu01, Cloud-Clone Corp., Katy, TX, USA; working dilution 1:1000), anti-AKT (9272, Cell Signaling Technology, Danvers, MA, USA; working dilution 1:750), anti-pAKT (sc-7985-R, Santa Cruz Biotechnology, working dilution 1:500), and anti-actin (Ab8227, Abcam, Cambridge, UK; working dilution 1:1000). The immunoreaction was performed at 4 °C overnight. After an exhaustive washing, the membranes were incubated with the peroxidase-conjugated anti-rabbit secondary antibody (Ab6721, Abcam; working dilution 1:3000) at room temperature for 1 h. The labeled protein bands were visualized with the molecular imager Versadoc MP imaging System (Bio-Rad, Hercules, CA, USA) using the Super Signal West Pico Plus substrate (ThermoFisher). The quantitative analysis of the bands was carried out with the ImageJ software with the normalization set to the actin band intensity, which was used as the internal control.

### 2.8. Statistics

The normality tests were performed with the SigmaPlot 11.0 software (SYSTAT, San Jose, CA, USA). For the Western blot experiments, the data were analyzed with the unpaired two-tailed Student’s *t*-test using GraphPad Prism 9 software (GraphPad, San Diego, CA, USA).

## 3. Results

### 3.1. Glycogen Synthesis by CFE-Treated HepG2 Cells

**Figure 1:**
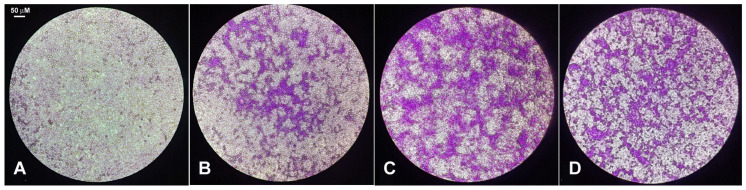
Representative micrographs of PAS staining in HepG2 cells grown in control condition (**A**) and after stimulation for 24 h with 10^−7^ M insulin (**B**), 2 μg CFE/mL (**C**), and 2 μg CFE/mL + 10^−7^ M insulin (**D**). Microscopic magnification = 10×.

In the first set of assays, the possible effect of the exposure of HepG2 cells for 24 h to the CFE on glucose metabolism was investigated through the evaluation of the intracellular accumulation of glycogen assessed by PAS staining. Figure 1 shows a panel of representative micrographs. As expected, in the presence of insulin a marked reaction could be observed compared to the untreated control. Of note, upon exposure to the CFE, the PAS staining appeared to further increase compared to insulin, whereas the co-treatment with insulin and the CFE was not associated with a greater glycogenesis than in the presence of the sole CFE.

### 3.2. Glucose Consumption and Glucose Uptake by CFE-Treated HepG2 Cells

**Figure 2:**
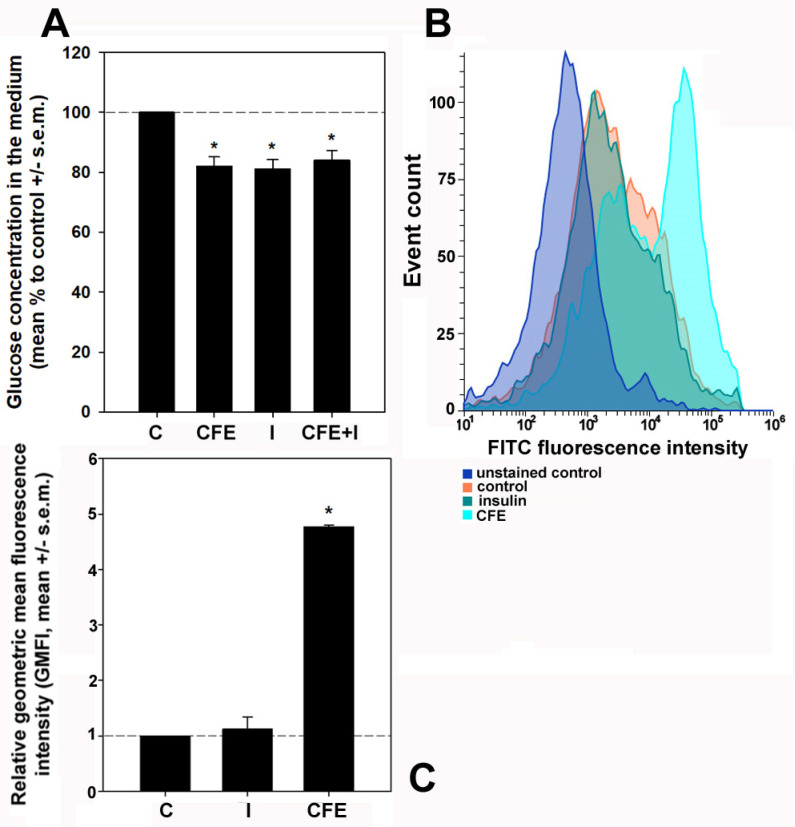
(**A**) Effect of the exposure for 24 h to 2 μg CFE/mL (CFE), 10^−7^ M insulin (I), and to both stimulators (CFE + I) on glucose concentration in the culture media of HepG2 cells vs. control (C). (**B**) Representative flow cytometric profiles of the uptake of 2-NBDG by HepG2 cells cultured for 1 h in control conditions or treated with 10^−7^ M insulin or 2 μg CFE/mL. The fully processed samples without the addition of 2-NBDG were assayed in parallel to control for the cellular autofluorescence (unstained control). (**C**) Bar graph showing the relative geometric mean fluorescence intensity (GMFI) of either control (C), insulin- (I), or CFE-treated (CFE) HepG2 cells. The cumulative data from the flow cytometry experiments were analyzed for the GMFI of each condition, and the GMFI of either treated cell population was divided by the GMFI of the controls to normalize the data. The error bars indicate the standard error of the mean (s.e.m.) of three independent measurements. * Normality test vs. control passed.

The impact of the CFE on glucose consumption in the presence or absence of insulin was monitored by evaluating glucose content in the culture media of control, insulin-treated, CFE-treated, and insulin/CFE co-treated HepG2 cells after 24 h of exposure. In addition, the 2-NBDG uptake assay was performed to complement the previous assay by determining the short-term glucose responsive action upon exposure. As reported in Figure 2, the treatment with the CFE for 24 h significantly decreased glucose concentration in the medium down to 82.1 ± 3% vs. the control, a value close to that obtained after insulin treatment (81.1 ± 3.2%). No synergistic effect was exerted by cell co-exposure to the CFE and insulin (84.1 ± 3.2%); therefore, in light of the irrelevance of both these and the PAS staining results in case of co-treatment, such an experimental condition was discarded in the following experiments. As a complement to the glucometric data, the flow cytometric evaluations of the internalization of 2-NBDG showed that after 1 h of treatment only the CFE induced a potent and immediate response leading to the stimulation of the uptake of the glucose analog up to +4.8 ± 0.03-fold vs. the control. Conversely, no significant short-term increase (+0.1 ± 0.2-fold) was found after the exposure to insulin.

### 3.3. Expression of Glucose Transporters and Upstream Regulators in CFE-Treated HepG2 Cells

**Figure 3:**
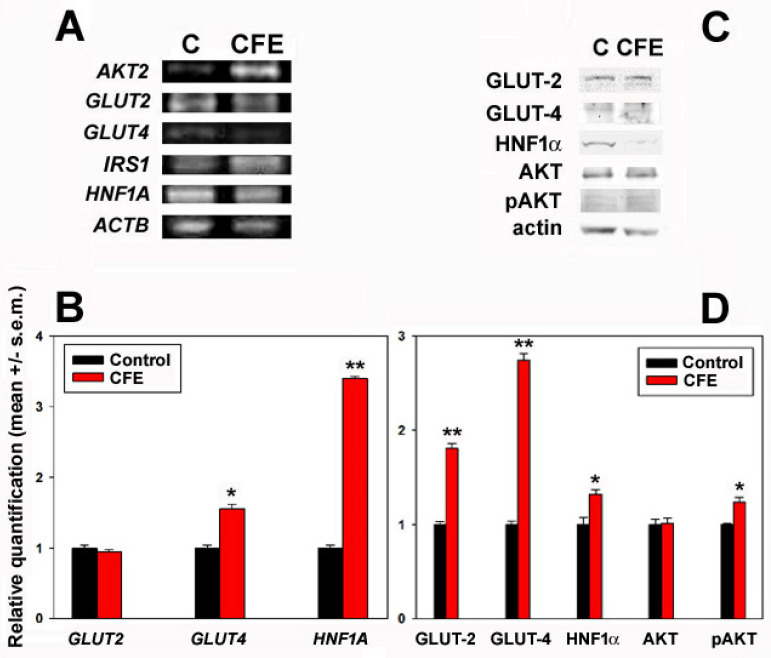
Conventional (**A**) and qRT (**B**) PCR analyses of the expression of genes coding for the glucose transporters and regulators by HepG2 cells grown in control conditions or exposed for 24 h to 2 μg CFE/mL (CFE). The panel in (**A**) is representative of three independent experiments. The bar graph in (**B**) depicts the relative quantification, normalized to *ACTB* expression, of three independent experiments. (**C**) Western blot analysis for the glucose transporters and their regulators in HepG2 cells grown in control conditions or exposed for 24 h to 2 μg CFE/mL (CFE). The whole Ponceau red- and immunostained blots are shown in Figure S1. The bar graph in (**D**) depicts the relative quantification obtained by band densitometry, normalized to actin, of three independent experiments of which the panel in (**C**) is representative. The values of the normalized band intensities are reported in Table S1 The error bars indicate the standard error of the mean (s.e.m.). * *p* ≤ 0.05; ** *p* ≤ 0.01.

The effect of cell exposure to the CFE on the, respectively, insulin-unresponsive and insulin-stimulated GLUT-2 and GLUT-4 transporters was studied at the gene expression and protein accumulation levels. In addition, three regulators were included in the mRNA and/or protein analysis. They were hepatocyte nuclear factor-1α (HNF1α), a GLUT-2 transcription factor [22,32], insulin receptor substrate-1 (IRS-1), and protein kinase B (AKT) with its activated form pAKT, the latter belonging to a pathway responsible for the surface translocation of GLUT-4 [25,33]. The panel in Figure 3A, representative of a triplicate experiment, shows the result of the conventional PCR assay indicating that (i) in control non-stimulated HepG2 cells, as expected [34], *GLUT-2* expression was apparently higher than that of *GLUT-4*, and (ii) about +6.1- and +2.4-fold increases in *AKT* and *IRS1* expression levels, respectively, were identified upon treatment by analysis of the amplicon intensity with the ImageJ software and normalization set to *ACTB*. Being undetectable by conventional PCR, the differential expression of *GLUT2*, *GLUT4*, and *HNF1A* was further searched through qRT-PCR. The bar graph in Figure 3B shows that the exposure to the CFE promoted the upregulation of *GLUT4* (about +0.66-fold vs. the control) and HNF1A (about +2.4-fold vs. the control), whereas no significant change was found for the expression level of *GLUT2*. The Western blot data on both glucose transporters, HNF1α and AKT, with its phosphorylated forms are illustrated in Figure 3C,D. The obtained results demonstrate that, (i) in line with the mRNA expression data, GLUT-4 was less abundant than GLUT-2 in control cells, and (ii) the CFE induced the increased accumulation of GLUT-4 (about +2.7-fold), HNF1α (about +1.3-fold), and, unexpectedly, also GLUT-2 (about +1.8-fold). Of note, the average pAKT/AKT ratio was also raised by about 14%.

### 3.4. Exposure of the GLUTs on the Plasma Membrane in CFE-Treated HepG2 Cells

**Figure 4:**
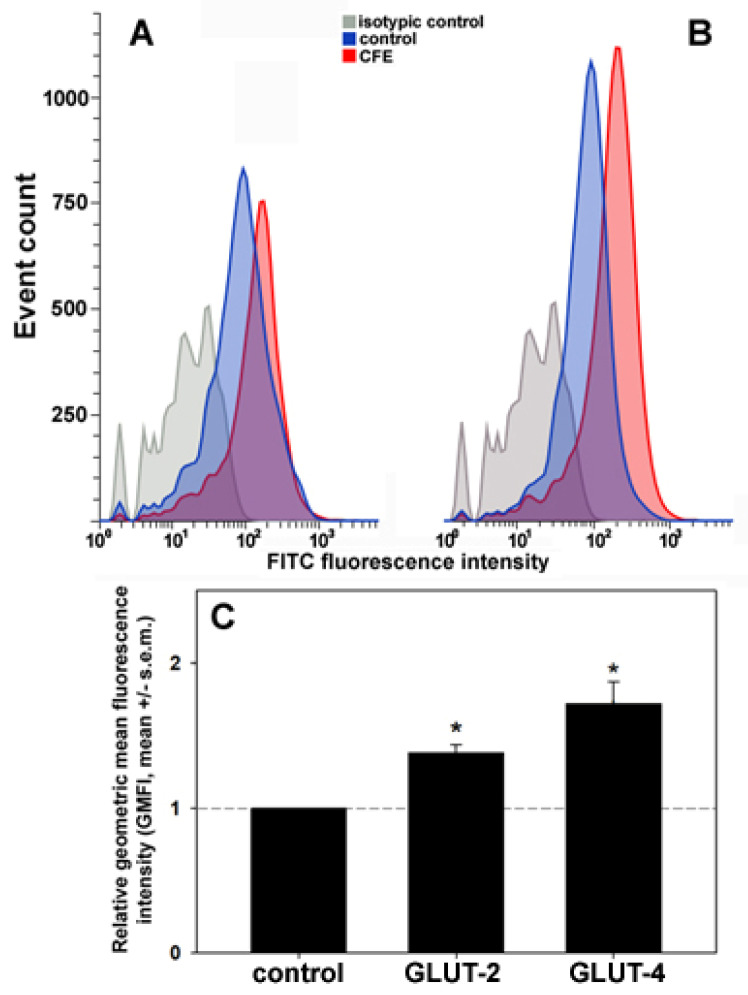
Representative flow cytometric analyses of immunostained GLUT-2 (**A**) and GLUT-4 (**B**) exposed on the surface of HepG2 cells grown for 24 h in control conditions or treated with 2 μg CFE/mL. An isotype control was included in the analysis. (**C**) Bar graph showing the relative geometric mean fluorescence intensity (GMFI) of GLUT-2 and GLUT-4 in CFE-treated cells normalized to control. The cumulative data from the flow cytometry experiments were analyzed for the GMFI of each condition, and the GMFI of either treated cell population was divided by the GMFI of the controls to normalize the data. The error bars indicate the standard error of the mean (s.e.m.) of three independent measurements. * Normality test vs. control passed.

Subsequently, to obtain more details on the effect of the extract on the glucose transport system, the extent of the exposure of GLUT-2 and -4 on the cell surface was assessed through the immunostaining of live HepG2 cells and flow cytometry. Figure 4 shows that (i) in control, unstimulated HepG2 cells the geometric mean fluorescence intensities (GMFI) of the flow data for the two transporters were analogous, and (ii) upon exposure for 24 h to 2 μg CFE/mL, a significant increase in the amount of GLUT-2 (about +38%) and, more prominently, GLUT-4 proteins (about +72%) exposed on the plasma membrane was detected.

## 4. Discussion

It is well known that holothurians are used as a traditional medical cure and nourishment for humans. Dealing with the anti-hyperglycemic effects, isolated compounds, such as the polysaccharides from *H. leucospilota* and *C. frondosa*, the AHG glycosaminoglycan from *A. japonicus*, the saponin from *H. thomasi*, and the eicosapentaenoic acid-enriched phosphatidylcholine from *C. frondosa*, have been proven to decrease blood glucose levels and improve the biochemical and histological markers of diabetes mellitus in experimental animal models. This occurred mostly via the activation of the signaling pathway regulating the exposure of GLUT-4 on the plasmalemma [35,36,37,38,39]. Also, an ethanolic extract from the golden sea cucumber *S. hermanii* was found to restore the glucose uptake and utilization in the muscles of diabetic rodents by improving GLUT-4 protein level [40].

To our knowledge, here we report the first evidence linking the treatment of liver cells with an *H. tubulosa*-derived extract with the modulation of glucose metabolism, unveiling a promising efficacy of the preparation as a potential anti-diabetic agent. It is acknowledged that diabetes mellitus-associated insulin resistance in the liver is characterized by the downregulation of the expression of GLUTs as well as a reduction in both the recruitment of the insulin-responsive glucose transporters, such as GLUT-4, from cytoplasmic vesicles and their positioning on the cell surface [41]. The translocation of GLUT-4 from the intracellular storage compartments to the external membrane is mediated mainly, although not exclusively, by the IRS-1/PI3K/pAKT pathway [25,42,43]. Of note, the activated pAKT is also responsible for enhanced glycogenesis through the upregulation of glycogen synthase, as shown in different experimental models including HepG2 cells [44]. On the other hand, the increase in the glucose uptake and glycogen storage in the liver may be also induced by enhancing the expression of the main transporter, i.e., GLUT-2 [22]. This was proven to be under the control of the homeodomain transcription factor HNF1α [45] and the IRS-1/AKT signalization pathway [42,46]. Our study shows that, by analogy with other natural substances, e.g., [22,24,42], the CFE induced the upregulation of *IRS1* gene expression and the increase in the transcription and translation levels of AKT and GLUT-4. Also, the phosphorylation level of AKT was enhanced by the treatment. On the other hand, the transcription and translation of *HNF1A* was likewise upregulated, whereas, although an increase in GLUT-2 protein could be observed, we were not able to find any upregulation of GLUT2 mRNA expression. As reported in other model systems [47,48], this suggests the existence of a mechanism of GLUT-2 protein regulation at the translation level or via post-transcription stabilization, which still needs to be determined. In parallel to the increased synthesis of GLUT-2 and -4 transporters, their significantly higher membrane translocation upon treatment was also found. Such molecular reprogramming conceivably improved the glucose response by HepG2 cells, leading to an evident increase in the glucose consumption/uptake and glycogen storage to levels comparable to those of insulin treatment.

Proteins are the main components of the cell-free coelomic fluid of echinoderms, and studies of its protein composition aimed at the identification of regeneration- and injury response-related factors have appeared in the literature [49,50]. We [16] have previously performed a proteomic profiling of the CFE from *H. tubulosa*, obtaining a final output of 115 forward and 20 reverse proteins and 321 unique forward peptides which were subjected to bioinformatic similarity search against different databases. We have re-evaluated this output searching for entries which might be associated with the extracellular glucose-lowering effect of the CFE on HepG2 cells and made the following considerations:

(i) No insulin or insulin analogs were found among the protein components of the CFE;
(ii) As already reported [16], the analysis of the mixture showed the presence of typical proteins of the exosomes which are conceivably kept intact by the method of preparation of the CFE and therefore can stimulate the observed effects upon fusion with HepG2 cells and intracellular transfer of their cargo;
(iii) Among the other protein signatures identified in the comprehensive analysis, three of them might be related to the increased recruitment and activation of GLUT-4. Their peptide sequences and the results of alignments selected on the basis of sorting by the best E value are reported in Table 2. In particular, they are the following:
  (1) Huntingtin-interacting protein 1 (HIP1), which is implicated in clathrin-mediated endocytosis and intracellular protein trafficking [51]. It is known that HIP1 interacts with the CHC22 clathrin isoform, expressed also in HepG2 cells [52], thereby maintaining its proper functioning which aims at the correct formation of the intracellular storage compartment for the GLUT-4 transporter [53]. The dysfunction of this mechanism has been linked to the onset of diabetes mellitus [54].
  (2) Small ubiquitin-like modifier (SUMO)/sentrin-specific protease 1 (SENP1), which is a cysteine protease that catalyzes the deSUMOylation of protein substrates, thereby controlling the intracellular localization and function of the targets [55]. Among them, the transcription factor HIF1α, which regulates the mobilization of GLUT-4-containing vesicles to the plasma membrane in skeletal muscle cells [56], is stabilized by deSUMOylation through SENP1 activity. Therefore, it might conceivably stimulate the increase in glucose uptake via the surface accumulation of GLUT-4 [57].
  (3) TBC1 domain family member 17 (TBC1D17), which is a Rab5 GTPase-activating protein. In myoblasts and skeletal muscle cells, the AMPK-induced phosphorylation of TBC1D17 leads to the activation of Rab5, which is known to recruit multiple molecules that intervene in GLUT-4 translocation [58].

**Table 2:** Glucose metabolism-associated proteins of the CFE from *H. tubulosa*.

Peptide Sequence	Sequence ID (Range)	Expected	Identities (%)	Positives (%)	Protein Description (Organism)
GRSAPSQGPNNGR	PIK47307.1 (459–471)	0.008	100	100	Putative huntingtin-interacting protein 1 isoform X3 (*Apostichopus japonicus*)
MSVSILDSMDTGKG	PIK42614.1 (182–195)	1 × 10^−4^	100	100	Putative sentrin-specific protease 1-like (*Apostichopus japonicus*)
KQVLTQAEGLVRE	PIK61193.1 (430–442)	0.002	100	100	Putative TBC1 domain family member 17 (*Apostichopus japonicus*)

## 5. Conclusions

In conclusion, we have produced in vitro data that demonstrate an evident increase in glucose consumption/uptake and glycogen storage after the exposure of HepG2 cells to the CFE. The molecular reprogramming underlying this effect involves the upregulation of (i) *IRS1* gene expression, (ii) the transcription and translation levels of HNF1α, AKT, and GLUT-4, (iii) the phosphorylation level of AKT, (iv) the synthesis of GLUT-2 protein, and (v) the translocation of GLUT-2 and -4 transporters onto the plasma membrane. From the perspective of an industrial application, the extraction of this biological matrix is much simpler and cheaper and also devoid of methodological issues compared to that of the polysaccharides obtained from the dried body wall of the sea cucumber which are endowed with a proven anti-diabetic activity “in vivo” [39]. Although the component(s) of the CFE responsible for the observed effects was (were) not isolated, the analysis of the proteomic profile nonetheless suggested the presence of some proteins that can seemingly enhance GLUT-4 activity. Of course, we cannot exclude the contribution of other trace proteins undetected in the proteomic analysis and/or non-protein water-soluble constituents, as well as the occurrence of synergic activities between the different components of the CFE.

Overall, the findings presented here add to the previously published anticancer effects of the extract [15,16], thus increasing interest in the biomedical implementation of this sea cucumber-derived preparation. Future work will be aimed at identifying the substance(s) responsible for the reported effect and to a more in-depth evaluation of the underlying molecular mechanism(s) responsible for the positive impact of CFE on glucose metabolism. Given the great need for developing alternative treatment options against diabetes mellitus, the aqueous extract of the coelomic fluid of *H. tubulosa* deserves interest for the development of novel treatment agents against this disease and beneficial supplements for the formulation of functional food.

## Figures and Tables

**Table 1:** Primers used for PCR amplification.

Gene (Primer)	Sequence (5′ → 3′)	Reference
*GLUT2* (sense)	GATGAACTGCCCACAATCTC	[31]
*GLUT2* (antisense)	CTGATGAAAAGTGCCAAGTG	
*GLUT4* (sense)	GTTAATCGGCATTCTGATCG	[31]
*GLUT4* (antisense)	GTGAAGACTGTGTTGACCAC	
*AKT2* (sense)	GCTAGGTGACAGCGTACCAC	[24]
*AKT2* (antisense)	GGCCTCTCGGTCTTCATCAG	
*IRS1* (sense)	TATCTGCATGGGTGGCAAGG	[24]
*IRS1* (antisense)	GGGTAGGCAGGCATCATCTC	
*HNF1A* (sense)	GAATGCATCCAGAGAGGGGT	[31]
*HNF1A* (antisense)	GTGGACCTTACTGGGGGAGA	
*ACTB* (sense)	GGAAGGTGGACAGCGAGGCC	[30]
*ACTB* (antisense)	GTGACGTGGACATCCGCAAAG	

## Data Availability

The data presented in the current study are available from the corresponding author upon request.

## Supplementary Materials

The following supporting information can be downloaded at: https://www.mdpi.com/article/10.3390/biology13060378/s1, Figure S1: Left: Images of the whole protein blots stained with Ponceau S and showing the molecular weight marker. Right: Images of the corresponding immunoblots whose trimmed inserts have been used for the panel in Figure 3C; Table S1: Mean intensity of the bands normalized to actin.
