# Supplementary material for: Modulation of Glucose Consumption and Uptake in HepG2 Cells by Aqueous Extracts from the Coelomic Fluid of the Edible Holothuria tubulosa Sea Cucumber
Source: Biology (Basel). 2024 May 25;13(6):378. doi: 10.3390/biology13060378 (PMC11201224; doi:10.3390/biology13060378)
Supplement: Supplementary file 1 [file biology-13-00378-s001.zip › biology-3002880-supplementary.pdf]

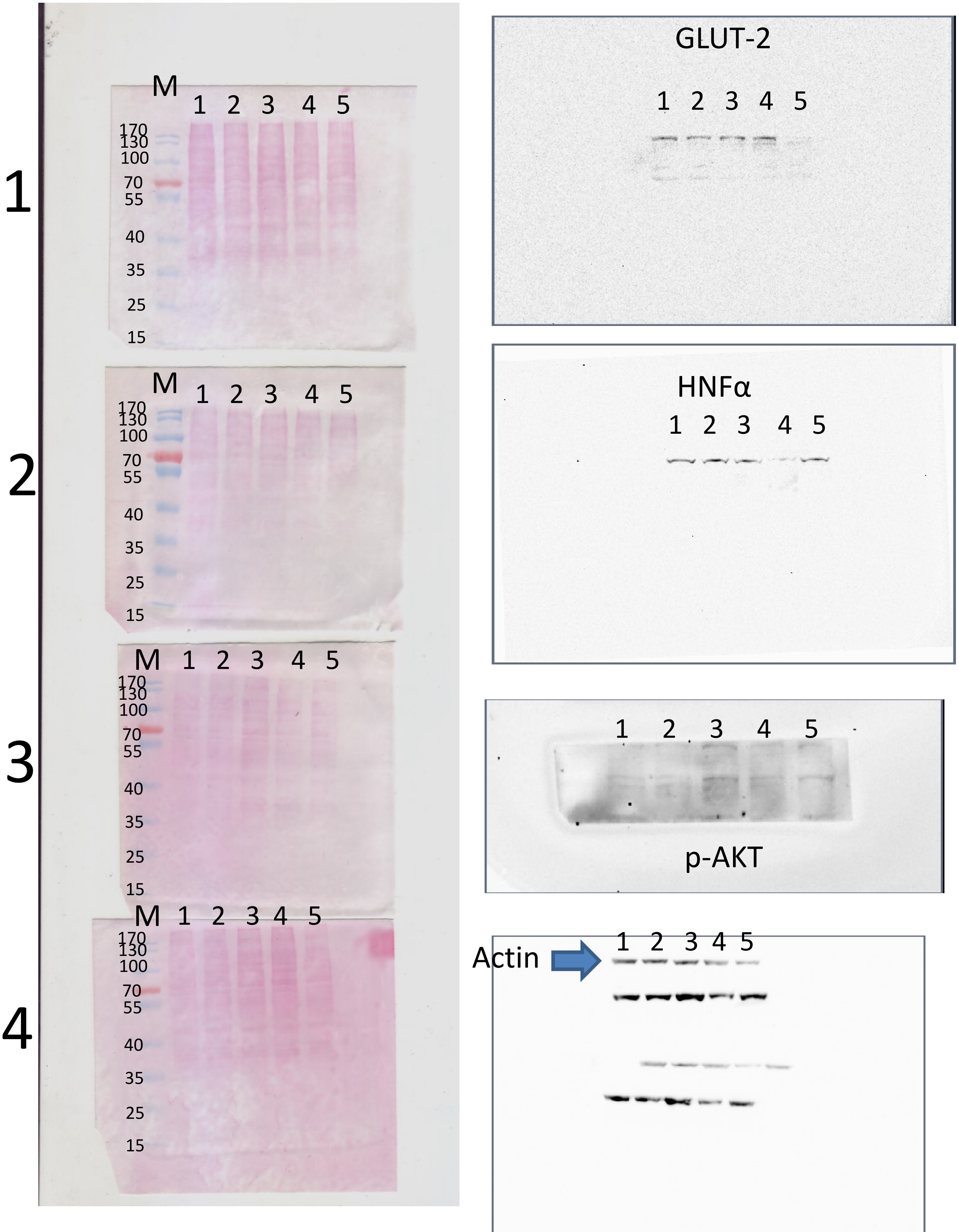

1
2
3
4
M 1 2 3 4 5
170
130
100
70
55
40
35
25
15
GLUT-2
1 2 3 4 5
HNFα
1 2 3 4 5
1 2 3 4 5
p-AKT
Actin
1 2 3 4 5

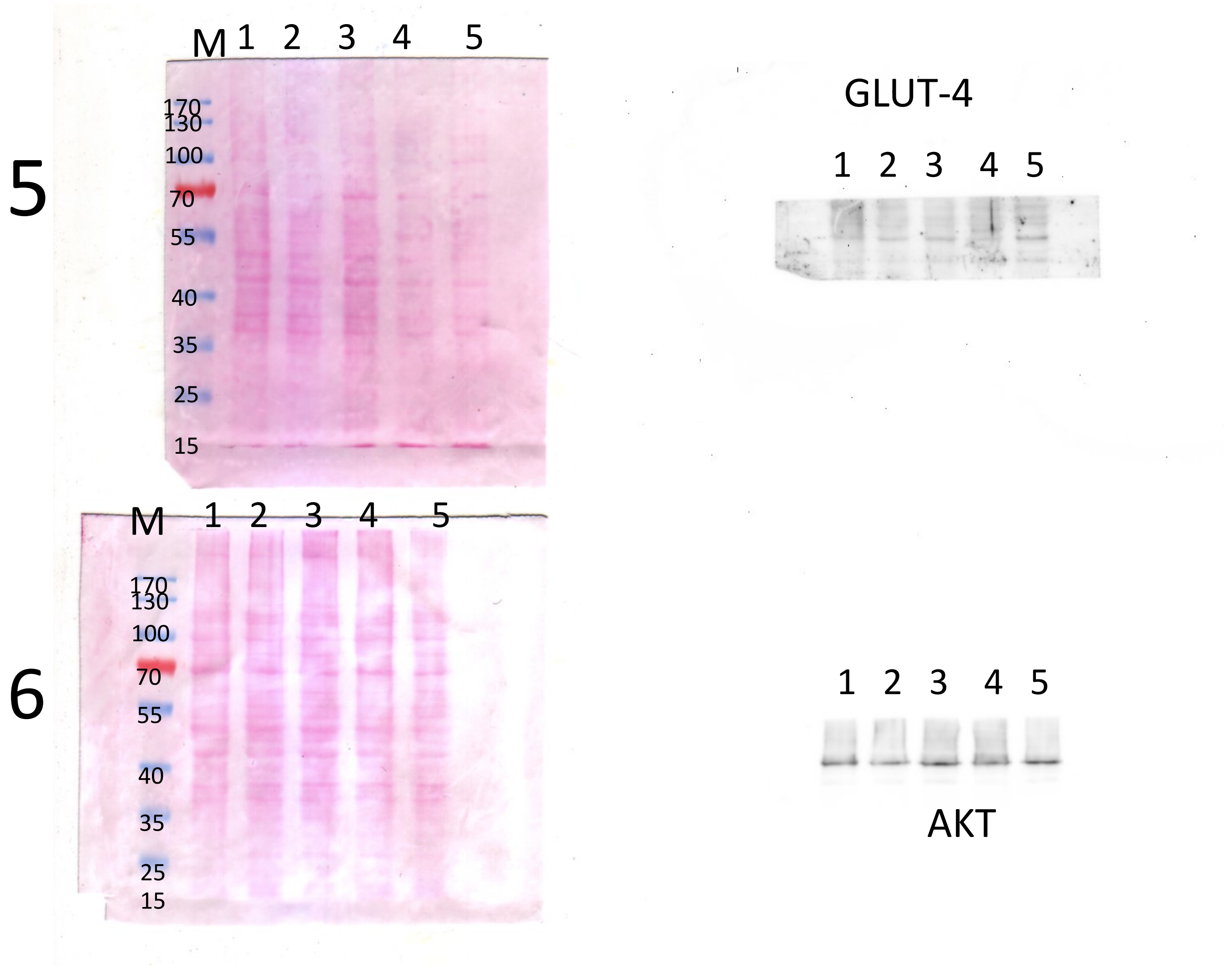

Figure S1: Left: Images of the whole protein blots stained with Ponceau S and showing the molecular weight marker. Right: Images of the corresponding immunoblots whose trimmed inserts have been used for the panel in fig. 3C. Control = sample 1, CFE = sample 4.

Table S1: Mean intensity of the bands normalized to actin

	**Glut 2**	**Glut 4**	**HNFα**	**AKT**	**pAKT**
Control	0.63	0.44	0.64	1.64	0.61
CFE	1.14	1.2	0.85	1.66	0.76
